# Identification of Main-Effect and Environmental Interaction QTL and Their Candidate Genes for Drought Tolerance in a Wheat RIL Population Between Two Elite Spring Cultivars

**DOI:** 10.3389/fgene.2021.656037

**Published:** 2021-06-17

**Authors:** S. M. Hisam Al Rabbi, Ajay Kumar, Sepehr Mohajeri Naraghi, Suraj Sapkota, Mohammed S. Alamri, Elias M. Elias, Shahryar Kianian, Raed Seetan, Ali Missaoui, Shyam Solanki, Mohamed Mergoum

**Affiliations:** ^1^Department of Plant Sciences, North Dakota State University, Fargo, ND, United States; ^2^Institute of Plant Breeding, Genetics, and Genomics, University of Georgia, Griffin, GA, United States; ^3^Department of Food Science and Nutrition, King Saud University, Riyadh, Saudi Arabia; ^4^USDA-ARS Cereal Disease Laboratory, University of Minnesota, St. Paul, MN, United States; ^5^Department of Computer Science, Slippery Rock University, Slippery Rock, PA, United States; ^6^Department of Crop and Soil Sciences, University of Georgia, Griffin, GA, United States; ^7^Department of Crop and Soil Sciences, Washington State University, Pullman, WA, United States

**Keywords:** drought tolerance, hard red spring wheat, quantitative trait loci, recombinant inbred line, marker-assisted selection

## Abstract

Understanding the genetics of drought tolerance can expedite the development of drought-tolerant cultivars in wheat. In this study, we dissected the genetics of drought tolerance in spring wheat using a recombinant inbred line (RIL) population derived from a cross between a drought-tolerant cultivar, ‘Reeder’ (PI613586), and a high-yielding but drought-susceptible cultivar, ‘Albany.’ The RIL population was evaluated for grain yield (YLD), grain volume weight (GVW), thousand kernel weight (TKW), plant height (PH), and days to heading (DH) at nine different environments. The Infinium 90 k-based high-density genetic map was generated using 10,657 polymorphic SNP markers representing 2,057 unique loci. Quantitative trait loci (QTL) analysis detected a total of 11 consistent QTL for drought tolerance-related traits. Of these, six QTL were exclusively identified in drought-prone environments, and five were constitutive QTL (identified under both drought and normal conditions). One major QTL on chromosome 7B was identified exclusively under drought environments and explained 13.6% of the phenotypic variation (PV) for YLD. Two other major QTL were detected, one each on chromosomes 7B and 2B under drought-prone environments, and explained 14.86 and 13.94% of phenotypic variation for GVW and YLD, respectively. One novel QTL for drought tolerance was identified on chromosome 2D. *In silico* expression analysis of candidate genes underlaying the exclusive QTLs associated with drought stress identified the enrichment of ribosomal and chloroplast photosynthesis-associated proteins showing the most expression variability, thus possibly contributing to stress response by modulating the glycosyltransferase (*TraesCS6A01G116400*) and hexosyltransferase (*TraesCS7B01G013300*) unique genes present in QTL 21 and 24, respectively. While both parents contributed favorable alleles to these QTL, unexpectedly, the high-yielding and less drought-tolerant parent contributed desirable alleles for drought tolerance at four out of six loci. Regardless of the origin, all QTL with significant drought tolerance could assist significantly in the development of drought-tolerant wheat cultivars, using genomics-assisted breeding approaches.

## Introduction

Hard red spring wheat (HRSW), comprising about 25% of the total United States wheat production, is unique for its high protein content (Vocke and Ali, 2013). However, this important crop often experiences drought, which is one of the main natural hazards harming wheat production worldwide (Araus et al., 2008). It regularly affects about 50% of wheat-producing areas (Pfeiffer et al., 2005). Drought refers to reduced accessible water in the soil and atmospheric conditions that cause plants to wilt or even die by losing water through transpiration. However, drought tolerance enables plants to yield satisfactorily under limited or periodic water-deficient conditions (Turner, 1979). Therefore, developing wheat cultivars with improved drought tolerance is the key to reduce yield loss due to water stress.

Drought tolerance in wheat can be achieved through developing cultivars capable of maintaining high water potential under drought conditions (Turner et al., 2001). Also, plants could escape from late-season drought through the development of early wheat cultivars (Araus et al., 2002). Understanding the genetics of drought tolerance in wheat is, therefore, a prerequisite to develop new adapted and drought-tolerant cultivars. Early research indicated that drought tolerance in crop plants is quantitatively inherited, or controlled by many genes or gene complexes (Blum, 1988), which can in turn be traced through quantitative trait loci (QTL) mapping methods.

Breeders have frequent debates over the appropriate phenotypic approaches for QTL analysis (Alexander et al., 2012). Many morphological traits, such as root length, tillering, spike number per m^2^, grain number per spike, number of fertile tillers per plant, one thousand grain weight, peduncle length, spike weight, stem weight, awn length, and grain weight per spike, can be affected by drought (Blum, 2005). However, yield (YLD) stability under both drought-stressed and favorable environments has been proposed for the effective selection of drought-tolerant genotypes (Pinter et al., 1990). From a breeder’s perspective, YLD and yield-related traits comprise the best morphological traits to screen for drought-tolerant genotypes. Hence, the source of QTL related to drought tolerance and the contribution of favorable alleles to this trait from diverse cultivars including high-yielding but non-drought-tolerant needs to be clarified.

An efficient tool for dissecting the genetics of drought is needed as most of the QTL mapping studies conducted on drought tolerance in wheat have used low-resolution maps composed of only several hundred molecular markers (Kirigwi et al., 2007; Muchero et al., 2009; Peleg et al., 2009; Sayed, 2011; Alexander et al., 2012; Ibrahim et al., 2012b; Kumar et al., 2012; Malik et al., 2015). Because of the size of the bread wheat genome (∼17 Gb), greater marker coverage is needed to generate a dense genetic linkage map, which could help to identify tightly linked markers associated with genes controlling traits of interest (Kumar et al., 2016, 2019). This is very important for the successful introgression of target loci in marker-assisted selection (MAS) and/or genomic selection methods in breeding programs. Precise identification of QTL will also facilitate easier positional cloning of those QTL (Kumar et al., 2016). The Infinium iSelect 90K assay, with 81,587 transcriptome-based single-nucleotide polymorphisms (SNPs) (Wang et al., 2014), can be an excellent tool for investigating the genetic basis of drought tolerance in wheat. Therefore, in this study, the main objective was to decipher the genetics of drought tolerance in wheat in the northern region of United States using a recombinant inbred line (RIL) population derived from a drought-tolerant cultivar ‘Reeder’ (PI613586) and a high-yielding and non-drought-tolerant cultivar ‘Albany’. Additionally, it has been long speculated (particularly, at the International Wheat and Maize Center, CIMMYT with “Shuttle” breeding concept, engineered by Dr. N. Borlaug) that many genes contributing positively to increased yield do so under both stressed and non-stressed conditions, including water stress/drought. Therefore, this study aims to elucidate that concept as well. The knowledge and resources developed using multi-location phenotypic data and high-density genetics map in this study will play an important role in our efforts toward development of drought-tolerant wheat cultivars.

## Materials and Methods

### Plant Materials

The cultivars Reeder and Albany were used to develop a RIL population consisting of 149 lines. Reeder is a drought-tolerant HRSW cultivar released by the North Dakota Agricultural Experiment Station at North Dakota State University (NDSU) in 1999. It is a semi-dwarf cultivar best adapted to western North Dakota (ND), a semiarid region of the state. Reeder has good milling and baking qualities and also possesses resistance to the Upper Midwest races of stem (caused by *Puccinia graminis* f. sp. *tritici*), stripe (caused by *Puccinia striiformis* Westend f. sp. *tritici* Eriks & Henn), and leaf (caused by caused by *Puccinia triticina* Erikss.) rusts. The other drought-sensitive parent, Albany, is a HRSW cultivar developed by Trigen Seed LLC. It is a very high-yielding, semi-dwarf cultivar adapted to high-input management conditions and better adapted to the eastern area of the Northern Plains spring wheat region, where drought conditions are not prevalent. A single seed descent (SSD) method was used to advance the RIL populations to the F_8_ generation. The study also included the checks, ‘Glenn’ (Mergoum et al., 2006), ‘SY Tyra’ (AgriPro^®^ wheat variety, United States), ‘Faller’ (Mergoum et al., 2008), ‘Steele-ND’ (Mergoum et al., 2005b), ‘Alsen’ (Frohberg et al., 2006), ‘Mott,’ ‘Elgin’ (Mergoum et al., 2016), ‘RB07’ (Anderson et al., 2009), ‘Dapps’ (Mergoum et al., 2005a), ‘Prosper’ (Mergoum et al., 2013), ‘ND901CLPlus’ (Mergoum et al., 2009) (PI655233), ‘Velva’ (Mergoum et al., 2014), ‘SY Soren’ (AgriPro^®^ wheat variety, United States), ‘Duclair’ (Lanning et al., 2011), ‘ND819’ (an elite experimental line developed by the NDSU spring wheat breeding program), ‘Polaris’, ‘Saturn’, and ‘Granite’ (PI619072). The checks ND819, Dapps, and Steele-ND are tolerant to drought stress. The genotypes SY Soren, Glenn, Alsen, ND901CLPlus, Saturn, and Velva show moderate tolerance, whereas Granite, Elgin, RB07, Duclair, Prosper, Mott, Faller, and SY Tyra are well adapted to high rainfall regions and therefore are most likely more susceptible to drought.

### Field Experiments and Phenotypic Data Collection

The evaluation of agronomic performances of the parents, RIL population, and 18 checks was carried out under non-irrigated field conditions at different locations in ND. The plant material was evaluated at Prosper and Carrington in 2012, 2013, and 2014; Minot in 2012; Williston in 2013; and Hettinger in 2014. Prosper is located in the eastern region of ND (46.9630°N, 97.0198°W). Carrington is located in the east-central region of ND (47.4497°N, 99.1262°W). Minot sits between semiarid grassland in the west and central ND’s subhumid grassland (48.2330°N, 101.2923°W). Williston is located in northwestern ND (48.1470°N, 103.6180°W) and Hettinger in southwestern ND (46.0014°N, 102.6368°W). The total rainfall in Prosper during the 2012, 2013, and 2014 growing periods (seed sowing to ripening) was 120.1 mm, 269.9 mm, and 176.8 mm, respectively (Table 1). Carrington had total rainfall of 171.2 mm, 159.8 mm, and 190.5 mm during the 2012, 2013, and 2014 growing periods, respectively. Moreover, during the same growing periods, Minot, Williston, and Hettinger had total rainfall of 162.2 mm, 320.4 mm, and 200.3 mm, respectively (Table 1; North Dakota Agricultural Weather Network (NDAWN), 2015). The available soil moisture of the experimental sites based on soil types is presented in Table 1. Each experiment was conducted in a randomized complete block design (RCBD) with two replicates. In 2012 and 2013, each genotype was planted in a 2.44 m × 1.22 m plot containing seven rows 15.24 cm apart. The plot size was slightly larger in 2014, at 2.44 m × 1.42 m, with the same number of rows (seven), but separation of 17.78 cm between rows.

**TABLE 1:** Soil types, plant-available water (water-holding capacity of soil), and total rainfall for nine environments.

**Environments**	**Soil type**	**Plant-available water (mm water/30.48 cm soil)**	**Rainfall (mm)**	**Total water available (mm)**
Prosper 12	Fine silty loam	45.7–63.5	120.1	165.8–183.6
Carrington 12	Coarse loamy	19.1–31.7	171.2	190.3–202.9
Minot 12	Fine sandy loam	31.7–45.7	162.2	193.9–207.9
Prosper 13	Fine silty loam	45.7–63.5	269.9	315.6–333.4
Carrington 13	Coarse loamy	19.0–31.7	159.8	178.8–191.5
Williston 13	Fine sandy loam	31.7–45.7	320.4	325.1–366.1
Prosper 14	Fine silty loam	45.7–63.5	176.8	222.5–240.3
Carrington 14	Coarse loamy	19.0–31.7	190.5	209.5–222.2
Hettinger 14	Fine sandy loam	31.7–45.7	200.3	232.0–246.0

Each year, the phenotypic data were recorded for days to heading (DH), plant height (PH), YLD, grain volume weight (GVW), and thousand kernel weight (TKW) at each site. The DH were taken when more than 50% of the plants in the plot were heading. The PH was measured from base to tip excluding the awn for plants in the middle of the plot. YLD per plot was converted to yield/ha for further analysis. Similarly, kg/0.5-pint cup was converted to kg/m^3^ as the GVW for further analysis. The TKW was measured by counting 1000 kernels using a seed counter (Model U, International Marketing and Design Co., San Antonio, TX, United States) and weighed.

### Phenotypic Data Analysis

The statistical analysis system used for analyzing the phenotypic data was ANOVA Proc MIXED (SAS Institute, 2004). The RILs, their parents, and the checks were considered as fixed effects, whereas environments and blocks were considered as random effects. The mean values were separated using the *F*-protected least significant difference (LSD) value at the *P* ≤ 0.05 level of significance. Pearson correlations between traits for each environment were calculated using the SAS’s CORR procedure (SAS Institute, 2004). Only the locations whose data exhibited a low coefficient of variation (CV) value and a significant difference among entries are reported in this study.

### Genotyping and Linkage Map Construction

Genomic DNA from each genotype was isolated from lyophilized young leaves using the DNeasy Plant Mini Kit (Qiagen, Valencia, CA, United States, cat. no. 69106). This DNA was run on 0.8% agarose gel to check its quality. The NanoDrop 1000 spectrophotometer (NanoDrop Technologies Inc., Wilmington, DE, United States) was used to check the DNA concentration. The RIL population, parents, and checks were genotyped using the Illumina 90K iSelect wheat SNP assay in the Small Grains Genotyping Lab (USDA-ARS, Fargo, ND, United States). The genotyping module GenomeStudio V2011.1^1^ was used to analyze the SNP data.

Polymorphic markers between parental genotypes were identified. Out of polymorphic loci, we discarded markers which had (1) an allele frequency of < 0.4 for any of the parental genotypes, (2) inconsistent results in five replicates of each parental genotype, (3) overlapping clusters for RILs, and (4) > 20% missing data. The remaining markers were used for map construction using a combination of MapMaker 3.0 (Lander and Botstein, 1989) and CartaGène v.1.2.3R (de Givry et al., 2005) software. At first, five to nine polymorphic markers from each chromosome covering the whole genome were selected as anchors based on available mapping information in multiple populations (Wang et al., 2014). Using MapMaker 3.0 (Lander and Botstein, 1989) and the anchor markers, 10,657 polymorphic markers were placed onto 21 wheat chromosomes using a minimum LOD score of 5.0 and a maximum distance of 40 cM. The linkage maps were then developed using CartaGène V.1.2.3R (de Givry et al., 2005). The details are described elsewhere (Kumar et al., 2012; Seetan et al., 2013, 2014). Briefly, the process starts with removal of identical markers. Then, initial maps are created using the “build” command, starting with the pair of most strongly linked markers. The remaining markers are then inserted incrementally. Then, the map is enhanced using “greedy search.” The next step uses genetic and simulated annealing algorithms for local improvement. In the final step, a fixed-length sliding window was applied to try all permutations within the window size to identify the map. Kosambi’s mapping function (Kosambi, 1944) was used to determine the genetic distance among markers on the linkage groups.

### QTL Mapping and Candidate Gene Identification

Composite interval mapping (CIM) was used to identify QTL for each trait in each environment as well as across environments (AE) using QTL Cartographer V2.5_011 (Wang et al., 2012). In QTL Cartographer, Model 6 (standard model), forward and backward regression, five control markers (co-factors), window size of 10 cM, and walk speed of 1 cM were used. A total of 1,000 permutations were used to determine the LOD threshold for identifying the significant QTL. Confidence intervals (CI) were estimated by the ± 2 LOD (from the peak) method. The QTL with overlapping CIs or QTL located within 10-cM regions were considered as the same QTL. Only the significant QTL detected (those above the threshold LOD score) were included in this study. If any such QTL were identified with an LOD score below the threshold, but > 2.5 in other environments, the QTL were also included in the results as supporting information. The QTL identified in at least two environments or associated with at least two traits were also reported in this study. The QTL regions were drawn using the Mapchart 2.3 program (Voorrips, 2002). Map locations of the associated markers were used to see if the QTL identified in this study have been reported in earlier studies. The sequences of the markers flanking each QTL were obtained from the T3/Wheat database (Blake et al., 2016), and their physical positions were extracted using the BLAST search against Chinese_Spring_IWGSC_RefSeq1.0 (Alaux et al., 2018; Appels et al., 2018). For each QTL, the position of flanking markers was used to determine the underlying block of high-confidence candidate genes and their annotated function^2^. *In silico* expression analysis for drought-specific QTLs (Table 2) was carried out in the Wheat expression browser expVIP (Borrill et al., 2016; Ramírez-González et al., 2018) dataset for drought and heat stress and PEG to stimulate drought and identify the expression variation of any repetitive functional class.

**TABLE 2:** Quantitative trait loci for drought tolerance in an RIL population derived from the cross between Reeder and Albany.

**QTL**	**Trait^†^**	**QTL region**	**Other associated traits**- I	**Env.^‡^**	**Position^§^**	**LOD^¶^**	**Additive effect**	***R*^2^ (%)**
*QDH.ndsu.2B.1*	DH	6	_	1, 2*, 3*	26.81–30.11	3.82	0.78	5.2
*QDH.ndsu.4A.2*	DH	13	GVW	1, 2, 3, 5, 6	133.91–143.11	9	−1.66	13.44
*QDH.ndsu.5A.2*	DH	16	_	1*, 2, 3	131.91–142.01	4.09	−0.92	6.22
*QDH.ndsu.5A.3*	DH	17	YLD, GVW	1, 3, 4, 5, 6	205.71–208.31	20.17	−2.84	38.36
*QDH.ndsu.5D2*	DH	20	GVW, YLD	2, 3, 4, 5, 6	11.91–20.91	15.16	2.29	29.93
*QTW.ndsu.2B*	GVW	7	YLD, HD	1, 2, 5, 6, M	84.31–95.61	8.02	−12.25	16.5
*QTW.ndsu.7B*	GVW	25	DH	1, 2, 3, M	29.11–40.11	8.95	−13.28	14.86
*QTKW.ndsu.2D.1*	TKW	8	_	2, 3*, M*	110.21–111.21	3.73	0.63	7.69
*QTKW.ndsu.6A*	TKW	21	_	1, 2, 3, 4*, 5, M	65.41–68.21	5.43	0.89	15.22
*QYL.ndsu.2B*	YL	7	GVW, HD	1,2, 3*, M	81.31–83.31	7.22	−209.44	13.94
*QYL.ndsu.7B*	YL	24	PH	1,2*	22.21–25.21	5.87	−178.75	13.6

- IPH = plant height, DH = days to heading, YLD = yield, GVW = grain volume weight, TKW = thousand kernel weight. ^‡^Env. = environment, 1 = Carrington 2012, 2 = Minot 2012, 3 = Prosper 2012, 4 = Carrington 2014, 5 = Hettinger 2014, 6 = Prosper 2014, 7 = mean across environments. ^§^ Position represents the peak point of the QTL interval. ^¶^ For log of odds (LOD) score. *The QTL in that environment was detected above a 2.5 LOD score, but below the threshold score.

## Results

### Climatic Conditions and Phenotypic Analyses

Climatic conditions that prevailed in 2013 were unusually variable (available water = 159.8 to 366.1 mm) causing high CV and were not conducive to drought, particularly in the western ND region (Williston), where rainfall was unusually high (Table 1). Additionally, the genotypes did not show significant differences for the agronomic traits in the 2013 trials (Supplementary Table 1). Therefore, given all these challenges and the criteria setup described earlier, only the data from the six environments planted in 2012 and 2014 were used in this study. For these two crop seasons, overall, total moisture available to the wheat crop, including soil residual water from snow, 2012 was considered as dry environment and 2014 was considered as control environment (Table 1). Total moisture available for wheat crop varied from 165.8 mm at Prosper in 2012 to 246 mm at Hettinger in 2014. Similarly, the DH data at Carrington in 2014 did not show a significant difference (Supplementary Table 1) and thus was discarded for further analysis. However, in the 2012 and 2014 field trials, significant differences among genotypes for most of the agronomic traits were observed.

**FIGURE 1:**
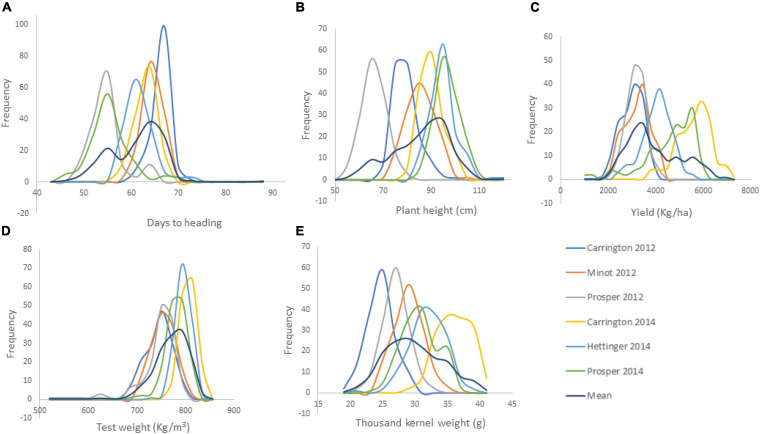
Frequency distribution of the agronomic traits of 149 RILs of the cross of Reeder and Albany (**A.** days to heading; **B.** plant height; **C.** yield; **D.** grain volume weight indicated here as test weight; and **E.** thousand kernel weight).

The RIL population showed continuous variation for all of the agronomic traits (Figure 1). Transgressive segregations in both directions were also observed among the RIL population for all traits in each and across environments (Supplementary Table 2). This shows that parents Reeder and Albany used to generate the RIL population were diverse and possess different favorable alleles for the studied traits. In particular, YLD means within each location and across the six environments varied significantly among RIL population with transgressive segregation. However, YLD did not differ significantly between the two parents Reeder and Albany with a slight YLD advantage to the later. For the RIL population, the mean YLD across the six environments varied from 2562.57 to 4461.78 kg ha^–1^. Meanwhile, YLD of parents Reeder and Albany varied from 3577.36 to 3800.93 kg ha^–1^, respectively. The check YLD mean was 3846.51 kg ha^–1^. Similar results of transgressive segregation among the RIL population were observed for all other traits, PH, HD, GVW, and TKW. Similarly, the parents Reeder and Albany did not differ for PH and GVW, while Reeder was significantly earlier and had higher TKW (Supplementary Table 2).

The heading date had a highly significant negative correlation with YLD, GVW, and TKW in all of the environments. Late-heading plants tended to be taller in two of the environments and also with the mean value of all the environments. Plant height did not show any significant association with any of the traits except DH. The higher-yielding genotypes had higher GVW in every environment. Similarly, higher-yielding genotypes had higher TKW in all environments except at Carrington in 2014. Again, the genotypes with high GVW tended to have high TKW in all environments except at Carrington in 2014 (Table 3).

**TABLE 3:** Correlation coefficients between five agronomic traits in the RIL population (Reeder × Albany) in six environments (Env.) and the overall mean across environments (M).

**Trait^†^ and Env.^‡^**	**PH**	**DH**		**YLD**		**GVW**
**DH**						
1	0.04ns	−	-	−	-	−
2	0.32***	−	-	−	-	−
3	0.07ns	−	-	−	-	−
4	.	−	-	−	-	−
5	0.13ns	−	-	−	-	−
6	0.31***	−	-	−	-	−
M	0.24**	−	-	−	-	−
**YLD**						
1	0.00ns	−0.58***	-	−	-	−
2	−0.03ns	−0.47***	-	−	-	−
3	0.18*	−0.38***	-	−	-	−
4	0.29***	.	-	−	-	−
5	0.06ns	−0.44***	-	−	-	−
6	−0.27***	−0.68***	-	−	-	−
M	−0.07ns	−0.59***	-	−	-	−
**GVW**						
1	−0.02ns	−0.62***		0.62***	-	−
2	−0.05ns	−0.57***		0.53***	-	−
3	−0.19*	−0.72***		0.51***	-	−
4	0.029ns	.		0.18*	-	−
5	0.01ns	−0.33***		0.29***	-	−
6	−0.21**	−0.55***		0.61***	-	−
M	−0.14ns	−0.6***		0.49***	-	−
**TKW**						
1	0.17*	−0.45***		0.56***		0.47***
2	0.2**	−0.33***		0.34***		0.45***
3	0.2*	−0.28***		0.23**		0.29***
4	0.19*	.		0.12ns		0.03ns
5	0.08ns	−0.48***		0.25**		0.36***
6	−0.06ns	−0.5***		0.47***		0.52***
M	0.13ns	−0.4***		0.29***		0.30***

*Significant at p < 0.05. **Significant at p < 0.01. ***Significant at p < 0.001 level ^†^PH = plant height, DH = days to heading, YLD = yield, GVW = grain volume weight, TKW = thousand kernel weight. ^‡^1 = Carrington 2012, 2 = Minot 2012, 3 = Prosper 2012, 4 = Carrington 2014, 5 = Hettinger 2014, 6 = Prosper 2014, M = Mean across environments.

### Genetic Linkage Map

Out of 81,587 SNPs markers in the Illumina iSelect 90K assay (Wang et al., 2014), 12,151 SNP polymorphic markers between parental genotypes were identified (Supplementary Table 3). After discarding unsuitable markers, 10,760 markers were eventually used for map construction. Out of the 10,760 markers selected for linkage mapping, 10,657 markers were mapped onto 28 linkage groups found on 21 wheat chromosomes (Table 4; Supplementary Table 3). The 10,657 markers represented 2,057 unique loci (19.3%), and 8,600 markers (80.7%) co-segregated with other loci. The B-genome contained the most number of markers, followed by the A-genome and the D-genome (Table 4). The number of markers on individual linkage groups ranged from 5 (1D1, 5D2) to 1,221 (2B), while for individual chromosomes, the number of markers ranged from 48 (chromosome 3D) to 1,221 (chromosome 2B) (Table 4). The average number of markers mapped per chromosome was 507.48, while the average number of unique loci per chromosome was 97.95.

**TABLE 4:** Distribution of markers across linkage groups in the genetic map developed using the Reeder × Albany RIL population.

**Linkage groups**	**No. of markers**	**No. of unique loci**	**Map length**	**Average map density**	**Average map density**
	
				**cM/marker**	**cM/locus**
1A	567	126	174.90	0.31	1.39
2A	439	101	223.50	0.51	2.21
3A	659	123	213.90	0.32	1.74
4A	560	114	218.90	0.39	1.92
5A	605	163	299.00	0.49	1.83
6A	590	117	176.70	0.30	1.51
7A	905	168	235.30	0.26	1.40
1B	629	86	107.50	0.17	1.25
2B	1221	160	181.80	0.15	1.14
3B	1115	213	250.20	0.22	1.17
4B	244	78	120.90	0.50	1.55
5B1	565	125	209.40	0.37	1.68
5B2	25	8.00	18.00	0.72	2.25
6B	426	101	158.10	0.37	1.57
7B	723	134	213.20	0.29	1.59
1D1	5	2	0.30	0.06	0.15
1D2	254	40	87.80	0.35	2.20
1D3	91	26	126.10	1.39	4.85
2D	653	46	180.40	0.28	3.92
3D	48	18	162.90	3.39	9.05
4D	53	23	129.90	2.45	5.65
5D1	25	8	47.50	1.90	5.94
5D2	5	4	24.90	4.98	6.23
5D3	130	21	31.50	0.24	1.50
6D1	10	5	3.00	0.30	0.60
6D2	23	19	44.50	1.93	2.34
6D3	22	6	4.00	0.18	0.67
7D	65	22	149.00	2.29	6.77
A genome	4,325	912	1,542.20	0.37	1.72
B genome	4,948	905	1,259.10	0.35	1.52
D genome	1,384	240.00	991.80	1.52	3.84
Whole genome	10,657	2,057	3,793.10	0.36	1.84

The 10,657 (2,057 loci) markers mapped in this study covered a total genetic map length of 3,793.1 cM, with an average distance of 0.36 cM between any two markers (Table 4). The A-genome chromosomes covered a total length of 1,542.2 cM, with an average distance of 0.37 cM between two markers. The B-genome had a total map length of 1,259.1 cM, with an average distance of 0.35 cM between two markers. The D-genome covered a total map length of 991.8 cM, with an average distance of 1.52 cM between two markers. Individually, chromosome 5A was the longest, with a total map length of 299 cM. Chromosome 6D was the shortest, with a total map length of 51.5 cM. Overall, the marker order was consistent with earlier studies on wheat genetic maps (Wang et al., 2014).

### QTL Analysis

#### YLD

Composite interval mapping for YLD identified six QTL located on six different chromosomes (Table 5; Supplementary Figure 1). Four of these QTL explained greater than 10% of PV and were considered as major QTL. The major QTL located on chromosome 2B had a phenotypic variation (PV) up to 13.94%; that on 5A had a PV up to 22.35%; and that on 5D had a PV up to 22.83%. All these three QTL were identified in three of the environments and in the overall mean and, thus, could be considered as consistent or stable QTL. The fourth major QTL on chromosome 7B was identified in one location and in the overall mean, explaining up to 13.6% of PV. The alleles for higher YLD for the QTL on chromosomes 5D, 2B, and 7B were contributed by the parent Albany, whereas the allele for the major QTL on chromosome 5A was contributed by Reeder (Table 5).

**TABLE 5:** Quantitative trait loci identified for the agronomic traits in an RIL population derived from the cross between Reeder and Albany.

**QTL and trait**	**QTL region**	**Other associated traits^†^**	**Env.^‡^**	**Position^§^**	**LOD^¶^**	**Additive effect**	**R^2^ (%)**
**Days to heading (DH)**							
*QDH.ndsu.2B.1*	6	_	1, 2*,3*	26.81−30.11	3.82	0.78	5.2
*QDH.ndsu.2B.2*	7	YLD, GVW	1	76.11	4.25	0.52	5.74
*QDH.ndsu.4A.1*	12	TKW	2	47.51	4.56	−0.66	8.24
*QDH.ndsu.4A.2*	13	GVW	1, 2, 3, 5, 6	133.91−143.11	9	−1.66	13.44
*QDH.ndsu.5A.1*	15	_	1*, 6	109.51−112.61	3.48	−0.61	4.12
*QDH.ndsu.5A.2*	16	_	1*,2, 3	131.91−142.01	4.09	−0.92	6.22
*QDH.ndsu.5A.3*	17	YLD, GVW	1, 3,4, 5, 6	205.71−208.31	20.17	−2.84	38.36
*QDH.ndsu.5D2*	20	GVW, YLD	2, 3, 4, 5, 6	11.91−20.91	15.16	2.29	29.93
*QDH.ndsu.7B*	25	GVW	1, 2, 3, 4, 5*, 6	27.41−31.11	10.25	1.43	17.41
**Plant height (PH)**							
*QPH.ndsu.2A*	5	_	1, 4*	128.41−133.11	3.60	1.49	7.68
*QPH.ndsu.2D*	9	TKW	1, 3, 4, M	151.11−165.71	7.31	2.04	17.2
*QPH.ndsu.3B*	10	_	3, 6*,M*	184.31−187.71	4.33	1.53	8.55
*QPH.ndsu.4A*	14	_	2*, M*	175.01−176.01	3.23	−1.70	6.73
*QPH.ndsu.5B1*	18	_	5,6	32.41−33.21	4.5	−1.81	9.01
*QPH.ndsu.6A*	22	_	2*, 3, 4	85.51−90.61	5.28	1.83	11.37
*QPH.ndsu.7B.1*	26	_	1, 3*, 6, M	129.41−130.31	4.94	1.54	9.44
*QPH.ndsu.7B.2*	24	YLD	4*, 5, M*	24.21−26.21	3.69	1.81	9.36
**Grain volume weight (GVW)**							
*QTW.ndsu.2A.1*	4	_	1, 2, 5	100.71−104.31	4.53	−7.73	8.16
*QTW.ndsu.2A.2*	3	TKW	4, 5, 6*,M*	80.11−82.11	7.14	−6.45	15.93
*QTW.ndsu.2B*	7	YLD, HD	1, 2, 5, 6, M	84.31−95.61	8.02	−12.25	16.5
*QTW.ndsu.4A*	13	DH	6	139.91	3.79	5.8	7.22
*QTW.ndsu.5A*	17	YLD, DH	3, 6	207.01	9.43	20.77	17.79
*QTW.ndsu.5D2*	20	DH, YLD	3, 6, M	11.91	12.38	−25.22	24.47
*QTW.ndsu.7B*	25	DH	1, 2, 3, M	29.11−40.11	8.95	−13.28	14.86

**QTL and trait**	**QTL region**	**Other associated traits^†^**	**Env.^‡^**	**Position^§^ (cM)**	**LOD^¶^**	**Additive effect**	**R^2^ (%)**

**Thousand kernel weight (TKW)**							
*QTKW.ndsu.1A*	1	_	4*, 6*	87.61–94.01	3.43	−0.77	7.08
*QTKW.ndsu.2A*	3	GVW	3,4, M	76.51–78.21	4.36	0.82	9.66
*QTKW.ndsu.2D.1*	8	_	2, 3*, M*	110.21–111.21	3.73	0.63	7.69
*QTKW.ndsu.2D.2*	9	PH	1, 4	155.31–155.61	4.06	0.72	8.47
*QTKW.ndsu.4A*	12	DH	3	58.81	6.82	0.84	14.18
*QTKW.ndsu.5B1*	19	_	1*, 5*	152.01–153.01	2.72	−0.69	5.61
*QTKW.ndsu.6A*	21	_	1, 2, 3, 4*, 5, M	65.41–68.21	5.43	0.89	15.22
*QTKW.ndsu.7A*	23	_	1*, 3*	53.71	2.58	0.49	5.36
**Yield (YLD)**							
*QYL.ndsu.1B*	2	_	3,5*, M*	64.21–71.91	3.99	−259.69	8.57
*QYL.ndsu.2B*	7	GVW, HD	1,2, 3*, M	81.31–83.31	7.22	−209.44	13.94
*QYL.ndsu.3B*	11	_	4*, M*	202.21–213.81	3.17	−189.99	7.3
*QYL.ndsu.5A*	17	DH, GVW	3, 6, M	198.61–206.51	11.12	192.14	22.35
*QYL.ndsu.5D2*	20	GVW, DH	3, 5*, 6, M	11.91–14.91	10.49	−466.60	22.83
*QYL.ndsu.7B*	24	PH	1,2*	22.21–25.21	5.87	−178.75	13.6

^†^PH = plant height, DH = days to heading, YLD = yield, GVW = grain volume weight, TKW = thousand kernel weight. ^‡^Env. = environment, 1 = Carrington 2012, 2 = Minot 2012, 3 = Prosper 2012, 4 = Carrington 2014, 5 = Hettinger 2014, 6 = Prosper 2014, M = mean across environments. ^§^ Position represents the peak point of the QTL interval. ^¶^ For log of odds (LOD) score. *The QTL in that environment was detected above a 2.5 LOD score, but below the threshold score.

#### GVW

Seven QTL located on six different chromosomes were identified for GVW (Table **5**; Supplementary Figure 1). Five QTL among them were considered as major QTL as they have explained PV higher than 10%. The QTL with the greatest effect (PV of up to 24.47%) was located on chromosome 5D and identified in two different environments and in the overall mean. The second major QTL, with up to 17.79% PV, was on chromosome 5A and identified in two of the environments. The major QTL on chromosome 2B had the third greatest and consistent effect as it was identified in four different environments, with a PV of up to 16.5%. The fourth major QTL was located on 2A (with a PV of up to 15.93%) and was identified in three of the environments and in the overall mean. A fifth major QTL on chromosome 7B, explaining up to 14.86% of PV, was identified in three different environments and in the overall mean. The alleles for a higher grain volume weight for the major QTL on chromosomes 5D, 2B, 2A, and 7B were contributed by the parent Albany. The allele for the remaining major QTL on chromosome 5A was contributed by Reeder (Table 5).

#### TKW

The eight QTL identified for TKW were located on seven different chromosomes (Table 5; Supplementary Figure 1). Only three among these with PV more than 10% were considered major QTL. The major QTL with the largest phenotypic effect (with a PV of up to 15.22%) was located on chromosome 6A; it also had a consistent effect as it was identified in five different environments and in the overall mean. The second major QTL was located on chromosome 4A, explaining 14.18% of PV, but it was identified in only a single environment. Another QTL explaining up to 9.66% of PV was located on chromosome 2A and identified in two different environments and in the overall mean. The alleles for increased TKW for the major QTL on 6A were contributed by the cultivar Reeder (Table 5).

#### DH

Nine QTL located on five different chromosomes were identified for DH. These QTL explained from 4.12 to 38.36% of PV (Table 5; Supplementary Figure 1). Four QTL explained > 10% of PV and, therefore, can be considered as major QTL. The QTL with the greatest and consistent effect for DH was identified on chromosome 5A in all of the environments except one and explained up to 38.36% of PV. The second major QTL was identified on chromosome 5D in all of the environments except one and explained up to 29.93% of PV. The third major QTL explained 17.4% of PV and was identified on 7B in all of the environments. The fourth major QTL was identified on chromosome 4A in all of the environments except one and explained up to 13.44% of PV. The alleles for reduced DH on 5A and 4A were contributed by the parent Reeder, while the alleles for reduced DH on the other two major QTL were contributed by the parent Albany.

#### PH

Eight QTL identified for PH were located on seven different chromosomes (Table 5; Supplementary Figure 1). Two of them were considered major QTL (PV > 10%). The QTL found on chromosome 2D had the largest effect, explaining up to 17.2% of PV. This QTL was identified in three different environments and in the overall mean. The second major QTL found on chromosome 6A was also identified in three different environments and explained up to 11.37% of PV. Besides these, three more QTL explained almost 10% of PV. Two of them were identified on chromosome 7B, and another one on chromosome 5B. The QTL in the QTL region 26 of chromosome 7B was identified in three environments and in the overall mean. Another QTL in the QTL region 24 of chromosome 7B was identified in two of the environments and in the overall mean. The QTL on chromosome 5B was identified in two environments only. The alleles for reduced PH for the abovementioned QTL on chromosomes 2D, 6A, and 7B were contributed by the parent Albany. The allele for reduced PH on chromosome 5B was contributed by the parent Reeder (Table 5).

#### Co-localized or Pleiotropic QTL

Co-localized QTL could be used for simultaneous improvement of more than one trait when the desirable alleles come from the same parent. A total of 38 QTL were identified in this study for five agronomic traits (Table 5; Supplementary Figure 1). Many of those QTL had overlapping confidence intervals (CI). The QTL with overlapping CI or located within 10 cM of each other were considered as the same QTL region. Overall, these 38 QTL were located in 26 different genomic regions on 13 different chromosomes. A total of 21 co-localized or pleiotropic QTL were located in nine genomic regions. Individual genomic regions were associated with two to three traits. Genomic region 7 was associated with DH, YLD, and GVW. The QTL for YLD (*QYL.ndsu.2B*) and GVW (*QTW.ndsu.2B*) had a major effect, whereas that for DH (*QDH.ndsu.2B.2*) had a minor effect. The genomic region 20 located on chromosome 5D also harbored major QTL for the same three traits. The desirable alleles in both regions (7 and 20) were contributed by the parent Albany. Meanwhile, Reeder contributed the favorable alleles for genomic region 17 on chromosome 5A which harbored the major QTL (*QDH.ndsu.5A.3*, *QYL.ndsu.5A*, and *QTW.ndsu.5A*) for the same three traits.

Six genomic regions harbored QTL for two traits. Genomic region 12 harbored QTL for TKW (*QTKW.ndsu.4A*) and DH (*QDH.ndsu.4A.1*). The QTL for DH had a minor effect, whereas the QTL for TKW had a major effect. Reeder contributed the desirable alleles in both cases. Genomic region 13 harbored QTL for DH (*QDH.ndsu.4A.2*) and GVW (*QTW.ndsu.4A*). The QTL for DH was a major QTL, while that for GVW was minor. Desirable alleles for both traits were contributed by Reeder. Genomic region 25 was also associated with DH (*QDH.ndsu.7B*) and GVW (*QTW.ndsu.7B*). Both QTL had major effects, with the desirable alleles contributed by Albany. The QTL for PH (*QPH.ndsu.2D*) and TKW (*QTKW.ndsu.2D.2*) were associated with genomic region 9. The QTL for PH had a major effect, while that for TKW had a minor effect. Desired alleles from the QTL were contributed by both t parents. Genomic region 24 harbored QTL for PH (*QPH.ndsu.7B.2*) and YLD (*QYL.ndsu.7B*), where both QTL had major effects and the desired alleles came from Albany. Genomic region 3 harbored QTL for GVW (*QTW.ndsu.2A.2*) and TKW (*QTKW.ndsu.2A*). Both QTL had major effects, and the desired alleles were also contributed by both parents (Table 5; Supplementary Figure 1).

#### Drought Tolerance QTL

A total of 11 consistent QTL, important for drought tolerance, were identified. Among these, six QTL were exclusively detected under drought-prone environments and the remaining five were major constitutive QTL (PV > 10%) identified in both water regimes (Table 2; Supplementary Figure 1). The QTL *QTW.ndsu.7B*, which is also associated with DH, had a major effect on GVW with a LOD score of up to 8.95. The QTL *QYL.ndsu.2B* and *QYL.ndsu.7B* had major effects on grain yield. The desired alleles from these three major QTL were contributed by Albany, the high-yielding and less drought-tolerant parent. The QTL *QDH.ndsu.2B.1*, which had a LOD score of up to 3.82, controlled 5.2% of PV for DH. The desirable allele for this QTL was also contributed by the parent Albany. Another minor QTL for DH, *QDH.ndsu.5A.2*, had an LOD score of up to 4.09; however, the desired allele was contributed by the resistant parent Reeder. The third minor QTL, *QTKW.ndsu.2D.1*, controlled TKW with PV up to 7.69% and a LOD score of up to 3.73; Reeder contributed the desired allele.

#### Candidate Genes in Identified QTLs

We identified 3,862 genes (Supplementary Table 4; see Text footnote 2) with predicted functions in 26 reported QTLs controlling the abovementioned traits, by using the high-confidence annotated genes in Chinese_Spring_ IWGSC_RefSeq1.0. Some of these QTLS such as 3, 7, 17, and 20 are consistent in multiple environments (Table 5) for multiple traits; thus, underlaying genes in these QTLs possibly control the shared pathway, resulting in drought tolerance and phenotypic responses with associated traits. The candidate genes underlying the drought-specific QTLs were further mined using the wheatexp *in silico* expression analysis, and we identified 104 genes whose expression was reported to modulate during the vegetative and reproductive stage drought stress (Supplementary Table 5; Supplementary Figure 2). We also identified a glycosyltransferase (*TraesCS6A01G116400*) encoding gene and a hexosyltransferase (*TraesCS7B01G013300*) encoding gene as a single candidate present in QTLs 21 and 24, respectively. An enrichment of genes encoding for the large subunit of cytoplasmic and chloroplast ribosomal proteins and photosynthesis-associated genes were identified (Supplementary Table 5) in this expression-sorted 104 gene list, thus indicating the importance of these class of genes in drought stress.

## Discussion

### Linkage Map Construction

High-density single-nucleotide polymorphism (SNP) genotyping arrays explore genomic diversity and marker–trait associations very efficiently (Wang et al., 2014). The Infinium iSelect 90K assay (Wang et al., 2014) uses > 81,000 gene-associated SNPs to assess polymorphism in allohexaploid and allotetraploid wheat populations (Wang et al., 2014; Wu et al., 2015; Kumar et al., 2016; Liu et al., 2016; Sapkota et al., 2020). Use of this genotyping tool offers higher genome coverage and resolution in the dissection of wheat’s agronomic traits than those used in previous studies (Kirigwi et al., 2007; Muchero et al., 2009; Sayed, 2011; Alexander et al., 2012; Ibrahim et al., 2012a; Kumar et al., 2012; Milner et al., 2016). The main results related to marker density (0.36 cM/marker) or unique locus density (1.84 cM/locus) and genetic map length (3,793.1 cM) observed in this study agreed with the previous studies that used the 90K Infinium iSelect assay for genome mapping (Wang et al., 2014; Kumar et al., 2016). The A genome was found to be the longest, while the D genome was the shortest, which is also in agreement with previous studies (Kumar et al., 2016). The marker order strongly corresponded with several linkage maps developed using the Infinium iSelect 90K SNP assay, as well (Desiderio et al., 2014; Russo et al., 2014; Wang et al., 2014; Kumar et al., 2016; Sapkota et al., 2020).

Four of the chromosomes (1D, 5B, 5D, and 6D) had more than one linkage group. Chromosome 5B had two and chromosomes 1D, 5D, and 6D had three linkage groups. The probable reasons for the fragmentation could be the repeated elements that reside between gene-rich regions or the use of stringent mapping parameters (LOD score > 5 and distance < 40 cM) (Kumar et al., 2016). This fragmentation occurred mostly on the D-genome chromosomes as the Infinium iSelect 90K assay had a poor representation of the D genome (Wang et al., 2014). Further, the D genome is the newest inclusion in the hexaploid wheat genome (dating to around 10,000 years ago) and exhibits fewer polymorphisms than the other genomes according to previous studies (Dubcovsky and Dvorak, 2007).

### Use of Secondary Data to Assess Drought Conditions

According to Lanceras et al. (2004), drought can be assessed by variables like weather conditions, soil moisture, and crop conditions over a particular growing season. Rainfall data, which impacts soil moisture, was collected to assess drought conditions for this study. It was obtained from the NDAWN database^3^. The total amount of rainfall was collected from the date of planting to the date of plant physiological maturity in addition to soil residual water moister determined at planting (Table 1). The date of physiological maturity was calculated by adding 30 days to DH (Simmons et al., 1914). The 2012 crop season had less rainfall than 2014 in all of the environments, and therefore, the 2012 crop season can be considered as dry, whereas 2014 can be considered as normal season. The YLD data also support this categorization as all of the environments in 2012 had a lower YLD than in 2014.

### Use of Agronomic Data to Assess Drought Tolerance

Several studies suggested that drought tolerance can most effectively be incorporated into a breeding program by identifying QTL for YLD or YLD-related traits (Lanceras et al., 2004; Alexander et al., 2012). Yield is the trait of ultimate interest to breeders to develop adapted cultivars. In this study, YLD had a positive significant correlation with all studied traits except for DH, which was negatively correlated with YLD. In general, delayed DH gives a plant the opportunity to produce more photosynthates (the product of photosynthesis) and hence a higher YLD. However, in this study, YLD was higher for early (reduced DH) compared to late genotypes. This is usually well known in environments where terminal drought is common. In our study, snow accumulated during winter in the US North Central Plains. It is a major source of soil moisture in this region, and this soil moisture depletes with time. Therefore, the late genotypes (high DH values) were affected by drought, which resulted in reduced YLD. Except for PH, increased values were desirable for the rest of the agronomic traits as they have a positive correlation with YLD. A taller genotype (high PH) has the potential to produce more photosynthates and, therefore, should give more yield, but it often tends to lodge and compromises yield.

#### QTL for YLD

Grain YLD is considered the most significant trait to plant breeders. It is the result of all the phases of vegetative and reproductive development, therefore reflecting the contribution of all favorable alleles involved directly or indirectly in the formation of wheat kernels. It is therefore influenced by edaphic and aerial environments (Quarrie et al., 2006). QTL for YLD in wheat have been reported in several studies (McCartney et al., 2005; Quarrie et al., 2006; Kirigwi et al., 2007; Li et al., 2007, 2015; Maccaferri et al., 2008; Azadi et al., 2014; Cui et al., 2014; Edae et al., 2014; Gao et al., 2015; Narjesi et al., 2015; Milner et al., 2016). In this study, we revealed six QTL for yield, both major and minor, contributed by both parents of the RIL population, and the confirmation of the quantitative nature of inheritance of YLD. The QTL *QYL.ndsu.2B* on chromosome 2B at 81.31–83.31 cM identified in all the drought-prone environments is close to the QTL (*QGy.ubo-2B*) identified by Milner et al. (2016) in the same region. This QTL can be confirmed as drought-tolerant as it contributed to YLD in all environments with low rainfall. Narjesi et al. (2015) reported a YLD QTL at 8.5 cM on chromosome 5D. Our study also identified a QTL *QYL.ndsu.5D2* on the same chromosome, but at 11.91–12.91 cM on the second linkage group. However, considering the gaps between the linkage groups on the chromosome, the position of the QTL should be around the middle of the chromosome and therefore is most likely different from the one identified by Narjesi et al. (2015). Maccaferri et al. (2008) identified a YLD QTL (*QYld.idw-7B*) at 0 cM on chromosome 7B that could be the same QTL as *QYL.ndsu.7B* identified at 22.21–25.21 cM on the same chromosome. The closest reported QTL of *QYL.ndsu.1B* on chromosome 1B at 64.21–71.91 cM was *QYd-1B.1*, identified on the same chromosome at 23–28 cM (Cui et al., 2014). The QTL *QYld.abrii-3B.4* (Azadi et al., 2014) identified on chromosome 3B at 92.3 cM seemed to be different than the QTL *QYL.ndsu.3B* in this study. Similarly, the QTL *QYL.ndsu.5D2* and *QYL.ndsu.5A* are most likely to be novel QTL as no QTL were earlier reported around these positions.

#### QTL for TKW

Thousand kernel weight is one of the three major components of YLD. It is also important for grain quality, as larger and uniformly sized kernels are visually attractive, affecting GVW and commanding a higher market price (Ramya et al., 2010). Several studies have reported QTL related to wheat TKW (McCartney et al., 2005; Huang et al., 2006; Breseghello and Sorrells, 2007; Kuchel et al., 2007; Li et al., 2007, 2015; Zhang et al., 2008; Sun et al., 2009; Ramya et al., 2010; Azadi et al., 2014; Simmonds et al., 2014; Wei et al., 2014; Tadesse et al., 2015; Zanke et al., 2015; Kumar et al., 2016, 2019). This study revealed eight QTL having both major and minor effects for TKW, indicating its quantitative nature of inheritance. McCartney et al. (2005) identified the QTL *QGwt.crc-2A* occupying the same position as the QTL *QTKW.ndsu.*2A we identified in this study. The QTL *qTgw2A* (Wei et al., 2014) and *QTgw.abrii-4A.2* (Zhang et al., 2008) also occupied the same location. The QTL *QTgw.abrii-2D1.3* (Azadi et al., 2014) and *QTKW.ndsu.2D.2* identified in this study seemed to be the same QTL, occupying the same position on chromosome 2D. Similarly, the *QTKW.ndsu.4A* we identified on chromosome 4A seems to be in the same location as the QTL *QTgw.abrii-4A.2* (Azadi et al., 2014). The QTL *QTKW.ndsu.6A* was identified in all of the drought-prone environments, indicating its strong relationship with tolerance to drought. This QTL, however, occupied the same location as QTL*qTgw6A2* (Wei et al., 2014). Another QTL, *QTKW.ndsu.7A*, was also identified in the two drought-prone environments and could be comparable to *qTgw7A* (Wei et al., 2014) due to their proximity. The QTL *QTKW.caas-1A.1* (Li et al., 2015) and *QTKW.ndsu.1A* were most likely to be the same QTL since they were found in the same genomic region. However, there were no previous reports on QTL that corresponded with the QTL *QTKW.ndsu.2D.1* and *QTKW.ndsu.5B1*, indicating the probability that they are novel. The QTL *QTKW.ndsu.2D.1* could be very important for drought-tolerance breeding as it was identified in two of the drought-prone environments.

#### QTL for GVW

Grain volume weight is an important trait to wheat breeders as it impacts flour yield during milling (Rustgi et al., 2013). Quantitative trait loci for GVW were reported in several previous studies (McCartney et al., 2005, 2007; Huang et al., 2006; Narasimhamoorthy et al., 2006; Breseghello and Sorrells, 2007; Kuchel et al., 2007; Zhang et al., 2008; Sun et al., 2009; Rustgi et al., 2013; Hill et al., 2015; Tadesse et al., 2015; Kumar et al., 2016, 2019). In this study, we revealed seven QTL with both major and minor effects, indicating the quantitative nature of inheritance of GVW. The QTL identified in this study on chromosome 7B (*QTW.ndsu.7B*) at 29.11–40.11 cM was identified in all of the drought-prone environments, indicating its potential for drought tolerance. However, this QTL seemed to be close and similar to the QTL (*QTw.sdau-7B*) that Sun et al. (2009) identified. McCartney et al. (2005) identified a QTL, *QTwt.crc-2B*, linked with the marker *Xbarc183* at 96.7 cM on chromosome 7B that, according to the GrainGenes database, seemed to be the same as the QTL *QTW.ndsu.2B* identified in this study at 84.31–95.61 cM. This QTL was identified in two of the drought-prone environments. In the same study, McCartney et al. (2005) identified another QTL *QTwt.crc-5D*, between SSR markers *Xgdm63–Xwmc765* and positioned between 95 and 214.26 cM, according to the GrainGenes database. The QTL in this study, *QTW.ndsu.5D2*, could be the same as the later QTL as it is also located in the same genomic region. The nearest reported QTL to *QTW.ndsu.5A* was *QTw.hwwgr-5AS* (Li et al., 2016), which seemed to be a different QTL. The QTL *QTw.sdau-2A* (Sun et al., 2009) located between SSR markers *Xwmc181a* and *Xubc840c* seemed to be the same QTL as the QTL *QTW.ndsu.2A.2* identified in this study. However, in our study, we identified the QTL *QTW.ndsu.4A* QTL which does not correspond to any QTL reported previously and therefore is a novel QTL.

#### QTL for DH

As previously indicated, DH can be critical for drought tolerance, particularly in regions where late drought is prevalent. Therefore, many studies have been conducted and have identified many QTL for DH (Kato et al., 1999; Sourdille et al., 2000; Shindo et al., 2003; Xu et al., 2005; Griffiths et al., 2009; Alexander et al., 2012; Kamran et al., 2013; Bogard et al., 2014; Zanke C. et al., 2014; Guedira et al., 2016; Milner et al., 2016; Kumar et al., 2019). According to these studies, the genetic factors controlling DH are vernalization and photoperiod sensitivities, and earliness *per se* (Shindo et al., 2003). In general, vernalization divides wheat cultivars into two groups. Winter wheat needs cold temperatures (vernalization) to initiate flowering, while spring wheat does not need cold temperatures. Wheat is usually photosensitive and adapted to long-day conditions. Therefore, transitioning from vegetative to reproductive stages (spike emergence) is very late unless genotypes are exposed to long days. However, some genotypes are photoperiod insensitive and therefore can switch to reproductive stage (spike emergence and flowering) even in short days. On the other hand, earliness *per se* is the only environment-independent genetic factor controlling earliness (Shindo et al., 2003).

The present study revealed several major and minor QTL controlling DH, which confirms its quantitative nature of inheritance. Four major QTL (*QDH.ndsu.5A.3*, *QDH.ndsu.5D2*, *QDH.ndsu.7B*, and *QDH.ndsu.4A.2*) were found consistently under all drought conditions. The earliness *per se* QTL *QEet.ocs.5A.2* (Kato et al., 1999) on chromosome 5AL and the QTL identified in our study, *QDH.ndsu.5A.3* at 205.71–208.31 cM, occupy the same location and therefore may represent the same QTL. Similarly, the QTL *QDH.ndsu.4A.2* we identified on chromosome 4A may have corresponded with the QTL reported by McCartney et al. (2005). A relatively minor QTL, *QDH.ndsu.4A.1*, identified at 47.51 cM, could be the same as *QFlt.dms-4A.1* (Kamran et al., 2013). Sourdille et al. (2000) reported a QTL for earliness *per se* on chromosome 7BS, explaining 7.3% to 15.3% of PV. This and the QTL identified in our study on chromosome 7B could represent the same QTL due to their position in the same genomic region. In the same study (Sourdille et al., 2000), a QTL on the long arm of chromosome 5D for earliness *per se* was reported, and this may coincide with the QTL *QDH.ndsu.5D2* identified in this study.

#### QTL for PH

Plant height is considered crucial in wheat breeding programs as it relates to many important agronomic traits such as lodging resistance and a high harvest index. For example, the dwarfism gene from Nonglin-10 played a vital role in wheat breeding programs during the Green Revolution of the 1960s (Liu et al., 2011). This study showed that PH had a positive correlation with DH, whereas DH had a negative correlation with YLD. Therefore, it could be stated that reduced PH is desirable for higher YLD. Quantitative trait loci for PH have been reported in several previous studies (McCartney et al., 2005; Pushpendra et al., 2007; Liu et al., 2011; Huang et al., 2012; Zanke C.D. et al., 2014; Gao et al., 2015; Li et al., 2015; Narjesi et al., 2015; Milner et al., 2016; Singh et al., 2016). In this study, eight QTL were identified for PH, confirming the findings of Huang et al. (2012) who identified seven QTL for this trait. The QTL they identified on chromosome 2D at 144 cM and chromosome 5B at 64.67 cM could represent the same QTL identified in this study on chromosome 2D at 151.11–165.71 cM (*QPH.ndsu.2D*) and on chromosome 5B at 32.41–33.21 cM (*QPH.ndsu.5B1*), respectively. The QTL *QPH.ndsu.2D* for PH was identified in two drought-prone environments, indicating its potential to tolerate drought. Milner et al. (2016) identified a QTL (*Qph.ubo-7B*) for PH on chromosome 7B at 138.4 cM, which could be the same QTL (*QPH.ndsu.7B.1*) identified in this study on the same chromosome at 129.41–130.31 cM. This QTL was expressed in the drought-prone environments and thus could be useful for drought tolerance. Zanke C.D. et al. (2014) identified a QTL for PH at 93.5 cM on chromosome 6A that could be comparable with this study’s *QPH.ndsu.6A* at 85.51–90.61 cM on the same chromosome. This QTL was also identified in the two drought-prone environments. Previously, Zanke C.D. et al. (2014) identified another QTL at 36 cM on chromosome 7B for the same trait that could be comparable to QTL *QPH.ndsu.7B.2* identified in this study on the same chromosome at 24.21–26.21 cM. Similarly, they identified a QTL at 176.5 cM on chromosome 3B for PH, whereas this study identified a QTL at 184.31–187.71 cM for it on the same chromosome. They also identified a QTL at 117.2 cM on chromosome 2A, whereas in this study, we identified the QTL *QPH.ndsu.2A* on the same chromosome but at 128.41–133.11 cM.

### Pleiotropic QTL

The associations between traits in correlation studies could be justified by the co-localized or pleiotropic QTL. These co-localized QTL could be of great value to breeders if the desirable alleles come from the same parent. Desirable alleles from three genomic regions (7, 20, and 25) came from parent Albany (Table 5; Supplementary Figure 1). These QTL primarily have a major effect on YLD and YLD-related traits, making them even more important to crop improvement. The parent Reeder contributed all of the desirable alleles in three genomic regions (13, 17, and 24) (Table 5; Supplementary Figure 1). Most of these QTL also had the major effect on YLD and YLD-related traits. The remaining co-localized QTL from three genomic regions did not contain desirable alleles from the same parents.

### QTL for Drought Tolerance

The QTL identified on chromosome 7B (*QTW.ndsu.7B*) at 29.11–40.11 cM appears to relate strongly with drought tolerance as it was identified in all environments under drought conditions (Table 2). This QTL seems to coincide with a QTL that Sun et al. (2009) identified (*QTw.sdau-7B*) previously. The putative drought-tolerant QTL, *QYL.ndsu.7B*, was also identified very close to another major QTL, *QTW.ndsu.7B*, which is also associated with drought tolerance, indicating the importance of this genomic region to control drought tolerance. This result confirms the previous findings by Alexander et al. (2012), who found a QTL, *Qdt.ksu-7B*, located on chromosome 7B at 34.7 cM with significant effect on drought tolerance. Another putative major QTL, *QYL.ndsu.2B*, was detected in this study and corresponded with the QTL *QCrs*-, which was reported to have a negative effect on the trait of interest under both drought and control conditions (Ibrahim et al., 2012b). In the current study, however, the QTL was identified only in the environments with drought conditions. Similarly, the QTL *QDH.ndsu.5A.2* detected in our study occupied the same location as the previously reported QTL QHea + (Ibrahim et al., 2012a). In the latter study, the QTL *Qhea* + improved the trait of interest in both well-watered and drought conditions. However, in our study, *QDH.ndsu.5A.2* improved the trait of interest only under drought conditions. Ibrahim et al. (2012b) reported four QTL on chromosome 2D around 50 cM that improved the trait of interest under drought conditions. However, none of these reported QTL seemed to correspond with the QTL *QTKW.ndsu.2D.1* identified in this study. Therefore, this QTL can be considered as novel and more studies are needed to elucidate its importance in drought tolerance.

An additional QTL for DH, *QDH.ndsu.5A.3*, could be a constitutive QTL for drought tolerance since it was identified consistently in both drought and non-drought condition environments. This QTL could occupy the same genomic region as the earliness *per se* QTL, *Qeet.ocs.5A.2* (Kato et al., 1999). Similarly, another constitutive QTL for drought tolerance, *QDH.ndsu.5D2*, corresponded with a QTL for earliness *per se* located on the long arm of chromosome 5D (Sourdille et al., 2000). A constitutive QTL for drought tolerance through TKW was identified on chromosome 6A, which most likely represents the QTL *qTgw6A2* (Wei et al., 2014). Also, a constitutive drought-tolerant QTL, *QTW.ndsu.2B*, was identified for GVW, which could be the same QTL as *QTwt.crc-2B* (McCartney et al., 2005). All these QTL appear to play a crucial role directly or indirectly in drought tolerance.

### Candidate Gene Analysis

A total of 3,682 candidate genes were identified for the 26 QTLs (Table 5; Supplementary Table 4; see Text footnote 2) reported in this study. For QTL region 3, controlling the GVW and TKW traits in multiple environments, we identified only 21 candidate high-confidence annotated genes. Among these 21 identified candidate genes, adjacently located MYB transcription factor (*TraesCS2A01G097200*) and hexosyltransferase (*TraesCS2A01G097400*) could be the most important candidates due to the fact that in wheat the *MYB* transcriptional activator-coding gene, i.e., *TaMYB13*, was previously shown to be positively related with sucrose:sucrose 1-fructosyltransferase (*1-SST*) and sucrose:fructan 6-fructosyltransferase (*6-SFT*) mRNA levels, thus controlling the levels of fructan, which serves as a key water-soluble carbohydrate as a carbon source for grain filling (Xue et al., 2011). This locus also harbors the two NLR (nucleotide binding leucine-rich repeats) disease resistance proteins (*TraesCS2A01G096200*, *TraesCS2A01G096300*). NLRs act as immunity receptors in plants (Solanki et al., 2019; Walkowiak et al., 2020), which may indirectly play a role in the overall health of mature grain kernels. Another consistent QTL region was 7, affecting the YLD, GVW, and HD traits and harboring 978 annotated high-confidence genes (Supplementary Table 4; see Text footnote 2). YLD, GVW, and HD traits were also found to be associated with the QTL region 17 containing 114 annotated genes. QTL-20 contains 224 annotated genes (Supplementary Table 4; see Text footnote 2). However, there were no candidate genes present in the annotated genome between the QTL-25-associated flanking markers; thus, at this point we can only speculate that this region may control the trait of GVW and DH by harboring the trans-acting elements of gene regulation. The QTL region-24 correlated with YLD and PH traits and was found to be consistent in multiple environments. Interestingly, we only found one gene in QTL-24 annotated to encode for a hexosyltransferase (*TraesCS7B01G013300*), a homolog of *TraesCS2A01G097400*, a gene we proposed to be important for GVW and TKW traits controlled by QTL-3. Similarly, only one gene, *TraesCS6A01G116400*, in QTL-21 was found putatively encoding for a glycosyltransferase. Thus, these two genes possibly represent a major effect on drought stress. We also used an *in silico* expression analysis and short-listed 104 candidate genes among the all-underlying genes in drought-related QTL. The most frequent functional class in this differential regulation prioritized 104 candidate genes which associated with the ribosomal proteins and photosynthesis-associated proteins (Supplementary Table 5; Supplementary Figure 2), indicating a crucial role of this class of genes in drought stress.

## Conclusion

Understanding the genetic basis of drought tolerance in wheat is of immense value for developing drought-tolerant wheat varieties for world food security. In this study, a population developed from a cross between elite lines was used to elucidate the genetic factors involved in the control of drought tolerance in HRSW in northern United States. Multi-environment phenotypic data on yield-related traits, combined with a high-density Infinium 90K SNP-based genetic map, identified a total of nine QTL for DH, eight QTL for PH, seven QTL for GVW, eight QTL for TKW, and six QTL for YLD. The genetic dissection identified 11 consistent QTL related to drought tolerance in this population. These included six QTL exclusively associated with drought environments and five constitutive QTL (associated with both, drought, and normal conditions). Major QTL for drought tolerance were identified on chromosomes 7B, 2B, 5A, 5D, and 6A. One novel QTL for drought tolerance was identified on chromosome 2D. The ribosomal proteins and chloroplast photosynthesis-associated proteins were the major class found to be abundant in the 104 expression-sorted candidate genes in drought QTLs. Along with the single-candidate genes *TraesCS6A01G116400* and *TraesCS7B01G013300* at QTL 21 and 24, respectively, expression-sorted genes at six drought QTLs provide a valuable resource to breed for drought resistance. More importantly, the desirable alleles for several major loci were contributed by the high-yielding parent that was apparently susceptible to drought. This suggests that the high-yielding cultivars may contribute desirable QTL alleles for drought tolerance. Therefore, exploring high-yielding but seemingly drought-susceptible germplasms in the development of drought-tolerant cultivars is paramount. Although we were successful in identifying many major and minor QTL, future studies could focus on using other approaches (Wang et al., 2016) to detect possible minor effect QTL associated with wheat drought tolerance using different germplasms under different environments and types of drought.

The knowledge gained and closely linked markers associated with the major QTL and candidate genes identified in this study could be of immense value for understanding the genetic control of drought and can be valuable in marker-assisted breeding programs aimed at improving drought tolerance in wheat. The high-density maps that were developed also offer a better starting platform for the fine mapping and ultimately map-based cloning of major and stable loci identified in this study.

## Data Availability Statement

The raw data supporting the conclusions of this article will be made available by the authors, without undue reservation.

## Author Contributions

SR: data collection, analysis, and major write-up of the manuscript. AK: data analysis and manuscript write-up. SM: data collection and analysis. SSa: data analysis and write-up. MA: data and manuscript review. EE: data analysis and manuscript review. SK: data analysis and manuscript review. RS: data analyses. AM: data and manuscript review. SSo: data analysis and write-up. MM: conceptualized and designed the experiment, phenotypic data collection, and manuscript write-up and review. All authors contributed to the article and approved the submitted version.

## Conflict of Interest

The authors declare that the research was conducted in the absence of any commercial or financial relationships that could be construed as a potential conflict of interest.

## Footnotes

1 www.illumina.com

2 https://figshare.com/s/30a0fb5d21315fec1f01

3 https://ndawn.ndsu.nodak.edu/
