# Supplementary material for: Identification of Main-Effect and Environmental Interaction QTL and Their Candidate Genes for Drought Tolerance in a Wheat RIL Population Between Two Elite Spring Cultivars
Source: Front Genet. 2021 Jun 17;12:656037. doi: 10.3389/fgene.2021.656037 (PMC8249774; doi:10.3389/fgene.2021.656037)
Supplement: Supplementary file 1 [file Data_Sheet_1.zip › Supplementary file 1.DOCX]

Table S1. Analysis of variance of the RIL population for different agronomic traits evaluated in nine environments.

| Environment and sources^ᵻ^ | df | HD^ǂ^ | PH^§^ | YLD^¶^ | TW^#^ | TKW^ᵻᵻ^ |
| --- | --- | --- | --- | --- | --- | --- |
| Carrington 2012 |  |  |  |  |  |  |
| Treatment | 168 | 17.51*** | 4.41*** | 8.52*** | 13.13*** | 9.52*** |
| Error | 168 | 0.53 | 12.07 | 52396 | 124.22 | 0.93 |
| CV% |  | 1.11 | 4.52 | 8.12 | 1.51 | 4.04 |
| Minot 2012 |  |  |  |  |  |  |
| Treatment | 168 | 11.5*** | 2.11*** | 3.49*** | 8*** | 4.31*** |
| Error | 168 | 0.92 | 41.07 | 167980 | 158.39 | 2.43 |
| CV% |  | 1.51 | 7.7 | 13.56 | 1.69 | 5.51 |
| Prosper 2012 |  |  |  |  |  |  |
| Treatment | 168 | 18.67*** | 1.65*** | 2.49*** | 13.6*** | 10.5*** |
| Error | 168 | 1.49 | 39.67 | 131705 | 297.05 | 0.88 |
| CV% |  | 2.285 | 9.74 | 12.13 | 2.35 | 3.56 |
| Carrington 2013 |  |  |  |  |  |  |
| Treatment | 167 | 0.88 | 1.36* | 1.09 | 0.74 | 0.96 |
| Error | 155 | 8.21 | 20.82 | 273310 | 361.09 | 8.12 |
| CV% |  | 5.15 | 6.27 | 10.96 | 2.2 | 9.19 |
| Williston 2013 |  |  |  |  |  |  |
| Treatment | 168 | 1.24 | 0.97 | 1.13 | 0.78 | 1.07 |
| Error | 168 | 10.1 | 68 | 334357 | 182.46 | 6.83 |
| CV% |  | 5.12 | 14.7 | 22 | 1.64 | 8.67 |
| Prosper 2013 |  |  |  |  |  |  |
| Treatment | 168 | 0.91 | 1.3* | 1.14 | 0.7 | 0.74 |
| Error | 168 | 16.88 | 50.79 | 1577173 | 342.73 | 8.81 |
| CV% |  | 7.93 | 9.63 | 26.6 | 2.28 | 9.08 |
| Carrington 2014 |  |  |  |  |  |  |
| Treatment | 168 | 0.93 | 2.38*** | 1.75*** | 7.58*** | 5.09*** |
| Error | 168 | 12.75 | 22.7 | 553814 | 65.75 | 2.78 |
| CV% |  | 5.89 | 5.47 | 13.44 | 1.03 | 4.78 |
| Hettinger 2014 |  |  |  |  |  |  |
| Treatment | 168 | 3.91*** | 4.38*** | 4.31*** | 10.24*** | 8.05*** |
| Error | 168 | 4.97 | 16.11 | 187916 | 51.83 | 2.11 |
| CV% |  | 3.701 | 4.361661 | 11.1163 | 0.917296 | 4.629367 |
| Prosper 2014 |  |  |  |  |  |  |
| Treatment | 168 | 5.49*** | 4.49*** | 8.85*** | 9.18*** | 7.34*** |
| Error | 168 | 9.4 | 12.89 | 259910 | 95.36 | 2.6 |
| CV% |  | 5.66 | 3.83 | 11.32 | 1.27 | 5.35 |

*Significant at 0.05, ***Significant at 0.001 probability level

^ǂ^HD = days to heading, ^§^PH = height, ^¶^YLD = yield, ^#^TW = test weight, ^ᵻᵻ^TKW = thousand kernel weight

Table S2. Phenotypic performances of Reeder and Albany, RIL population and checks in six environments.

|  | | Parental lines |  |  |  | RIL population |  |  |  |
| --- | --- | --- | --- | --- | --- | --- | --- | --- | --- |
| Env.^ᵻ^ | | Reeder | Albany |  | Min | Max | Mean | Checks | LSD (0.05) |
|  | ……………………………….Plant height, cm………..………………………………. | | | | | | | | |
| 1 | | 77.47 | 68.58 |  | 67.31 | 97.79 | 76.82 | 78.32 | 6.81 |
| 2 | | 87.63 | 80.01 |  | 67.31 | 115.57 | 83.10 | 84.53 | 12.56 |
| 3 | | 62.23 | 57.15 |  | 52.07 | 77.47 | 64.19 | 69.36 | 12.34 |
| 4 | | 83.82 | 83.82 |  | 76.20 | 104.14 | 86.79 | 86.22 | 9.34 |
| 5 | | 92.71 | 88.90 |  | 80.01 | 119.38 | 92.07 | 91.86 | 7.87 |
| 6 | | 95.25 | 90.17 |  | 81.28 | 106.68 | 93.83 | 93.20 | 7.04 |
| M | | 83.19 | 78.11 |  | 74.30 | 101.39 | 82.80 | 83.91 | 9.33 |
|  | …………………………Days to heading, days………………………………………… | | | | | | | | |
| 1 | | 63.00 | 65.50 |  | 59.50 | 71.00 | 65.23 | 64.22 | 1.42 |
| 2 | | 61.50 | 64.00 |  | 57.50 | 69.50 | 63.27 | 63.00 | 1.88 |
| 3 | | 50.50 | 54.50 |  | 46.50 | 64.00 | 53.58 | 51.28 | 2.39 |
| 4 | | . | . |  | . | . | . | . | . |
| 5 | | 56.50 | 60.50 |  | 54.00 | 72.00 | 60.48 | 58.50 | 4.37 |
| 6 | | 50.50 | 55.50 |  | 45.50 | 87.50 | 54.59 | 50.47 | 6.01 |
| M | | 56.40 | 60.00 |  | 53.80 | 67.90 | 59.43 | 57.49 | 3.21 |
|  | ………………………………….Yield, Kg/ha………………………………………….. | | | | | | | | |
| 1 | | 2657.17 | 2984.96 |  | 1664.03 | 3913.01 | 2823.10 | 2793.97 | 448.65 |
| 2 | | 2864.88 | 2915.50 |  | 1761.58 | 4072.12 | 3043.44 | 2840.95 | 803.31 |
| 3 | | 2773.05 | 2558.44 |  | 1920.18 | 4121.90 | 2996.84 | 2996.80 | 711.31 |
| 4 | | 4490.20 | 6080.77 |  | 3650.85 | 7016.44 | 5457.19 | 5026.72 | 1458.61 |
| 5 | | 3791.92 | 3881.20 |  | 1610.86 | 5366.24 | 3864.81 | 4194.71 | 849.65 |
| 6 | | 4886.96 | 4384.69 |  | 654.42 | 6078.55 | 4416.39 | 5225.90 | 999.23 |
| M | | 3577.36 | 3800.93 |  | 2562.57 | 4461.78 | 3766.96 | 3846.51 | 878.46 |
|  | …………………………………..Test weight, Kg/m^3^_……………………………………………………….._ | | | | | | | | |
| 1 | | 722.47 | 747.17 |  | 662.17 | 798.93 | 733.79 | 750.45 | 21.85 |
| 2 | | 773.14 | 747.53 |  | 690.14 | 807.10 | 743.76 | 757.54 | 24.67 |
| 3 | | 748.62 | 773.87 |  | 501.81 | 790.76 | 731.27 | 753.48 | 33.78 |
| 4 | | 780.59 | 799.66 |  | 743.54 | 827.45 | 794.53 | 794.64 | 15.89 |
| 5 | | 799.84 | 784.95 |  | 720.84 | 818.91 | 784.07 | 790.59 | 14.11 |
| 6 | | 785.67 | 783.86 |  | 687.06 | 809.65 | 769.87 | 785.99 | 19.14 |
| M | | 768.39 | 772.84 |  | 678.61 | 803.02 | 759.55 | 772.11 | 21.57 |
|  | |  |  |  |  |  |  |  |  |

|  | Parental lines |  |  | RIL population |  |  |  |
| --- | --- | --- | --- | --- | --- | --- | --- |
| Env^ᵻ^ | Reeder | Albany | Min | Max | Mean | Checks | LSD (0.05) |
| ……………………Thousand kernel weight, g……………………………………….... | | | | | | | |
| 1 | 25.25 | 22.00 | 18.00 | 29.00 | 23.71 | 25.56 | 1.89 |
| 2 | 32.25 | 24.00 | 23.25 | 34.25 | 28.10 | 29.79 | 3.05 |
| 3 | 27.00 | 25.50 | 20.75 | 32.00 | 26.18 | 28.26 | 1.84 |
| 4 | 35.00 | 25.50 | 27.25 | 40.00 | 35.12 | 35.56 | 3.27 |
| 5 | 35.00 | 29.50 | 21.00 | 38.00 | 31.09 | 33.47 | 2.85 |
| 6 | 33.75 | 29.00 | 22.25 | 37.00 | 29.80 | 33.25 | 3.16 |
| M | 31.38 | 25.92 | 22.63 | 34.38 | 29.00 | 30.98 | 2.68 |

^ᵻ^1 = Carrington 2012, 2 = Minot 2012, 3 = Prosper 2012, 4 = Carrington 2014, 5 = Hettinger 2014, 6 = Prosper 2014, M = Mean across environments

**1A (2)**

**1A (1)**

**1B (1)**

**1B (2)**

**1D1**

**1D2**

**1D3**

**2A (1)**

**2A (2)**

**2B (2)**

**2B (1)**

**2B (3)**

**2D**

**3A (1)**

**3A (2)**

**3B (1)**

**3B (2)**

**3B (3)**

**3D**

**4A (2)**

**4A (1)**

**4B (1)**

**4B (2)**

**4D**

**5A (1)**

**5A (2)**

**5A (3)**

**5B1 (1)**

**5B1 (2)**

**5B3**

**5D1**

**5D2**

**5D3**

**6A (1)**

**6A (2)**

**6B (1)**

**6B (2)**

**6D1**

**6D2**

**6D3**

**7A (1)**

**7A (2)**

**7A (3)**

**7B (1)**

**7B (2)**

**7D**

**Fig. S1**. Complete genetic linkage maps of the RIL population derived from theReeder and Albany cross. The location of the QTL associated with agronomic traits are shown with a line on the right side of the linkage maps.


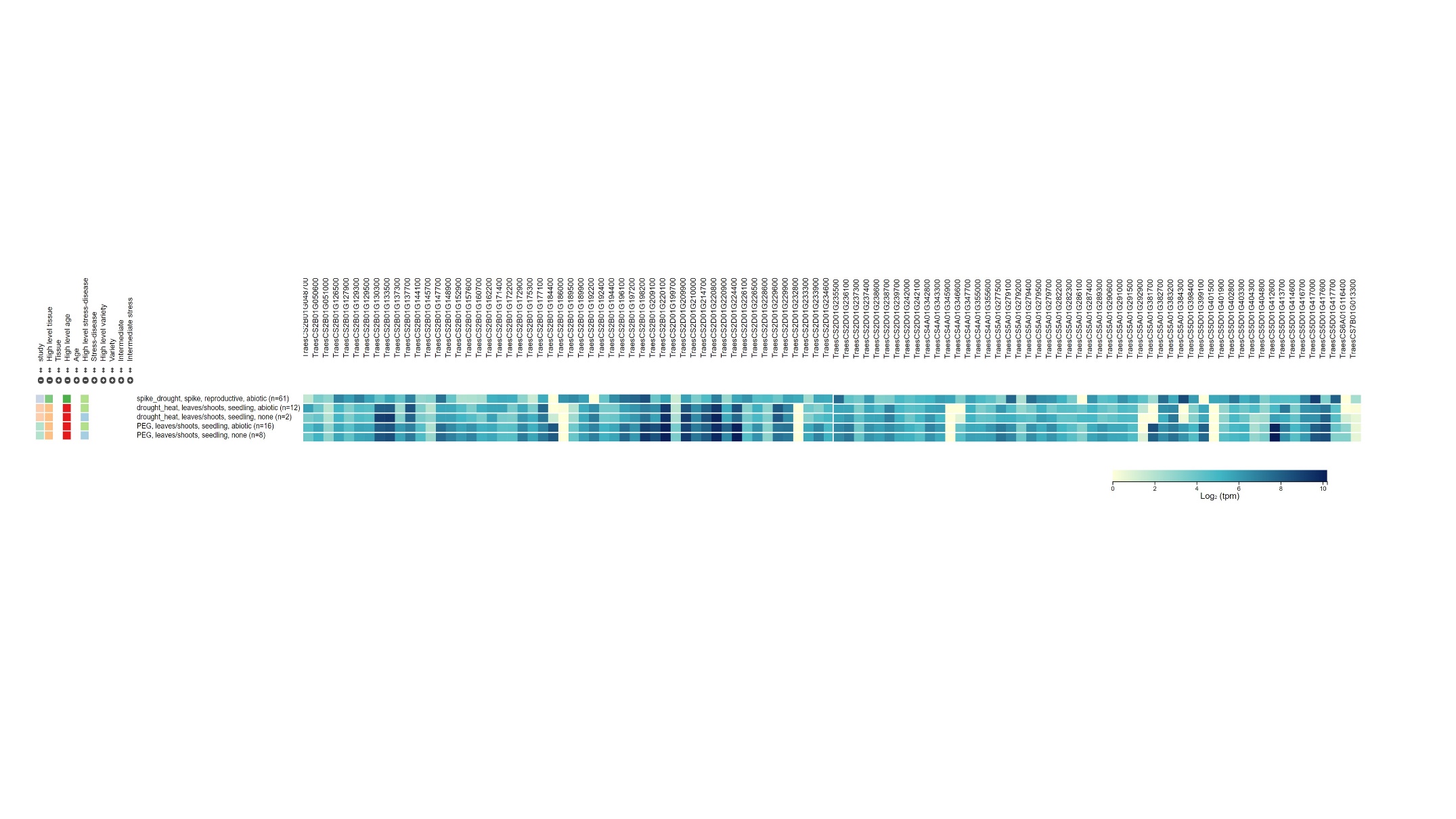


**Fig. S2:** Candidate gene analysis underlying drought specific QTL using the wheatexp *in silico* expression analysis. A total of 104 candidate genes were mined for drought related traits in wheat.
